# Magnetic Solid-Phase Extraction Based on C18 Nanoparticles for the Determination of Pesticides in Aquaculture Water Samples

**DOI:** 10.3390/molecules30092076

**Published:** 2025-05-07

**Authors:** Margarita Kapsi, Vasileios Sakkas, Vasiliki Boti, Triantafyllos Albanis

**Affiliations:** 1 Laboratory of Industrial Chemistry, Department of Chemistry, University of Ioannina, 45110 Ioannina, Greece; vboti@uoi.gr (V.B.); talbanis@uoi.gr (T.A.); 2 Institute of Oceanography, Hellenic Centre for Marine Research, 70014 Gournes, Greece; 3 Department of Agriculture, Hellenic Mediterranean University, 71410 Heraklion, Greece; 4 Laboratory of Analytical Chemistry, Department of Chemistry, University of Ioannina, 45110 Ioannina, Greece; vsakkas@uoi.gr

**Keywords:** chemometric tools, experimental design, magnetic octadecylsilane nanoparticles, pesticides, aquaculture waters

## Abstract

In this study, C18-functionalized magnetic silica nanoparticles (Fe_3_O_4_@SiO_2_@C18) were used as adsorbents for the magnetic solid-phase extraction (MSPE) of organic contaminants commonly applied to aquaculture water (organic booster biocides, herbicides, and insecticides) followed by Gas Chromatography coupled to Mass Spectrometry (GC–MS). The extraction conditions and efficiency of the nanoparticles for the determination of ten pesticides (atrazine, ethoxyquine, chlorothalonil, chlorpyriphos methyl, methyl parathion, chlorpyriphos, resmethrin, λ-cyhalothrin, permethrin, and irgarol) were thoroughly investigated. Several experimental parameters affecting the extraction efficiency such as the amount of sorbent, extraction time, and elution time were optimized by employing experimental designs as response surface methodology. Validation experiments showed that the average recoveries of target analytes were in the range of 60% to 99%. The optimized method exhibited good linearity (R^2^ > 0.9901) and satisfactory precision (Relative Standard deviations, RSDs < 15%). The method detection limits ranged between 1.9 ng L^−1^ and 62 ng L^−1^. Finally, the MSPE method was successfully applied to aquaculture water samples collected from the Thesprotia region (N.W. Greece), Thermaikos Gulf (N. Greece) and Butrint (S.W. Albania).

## 1. Introduction

Currently, intensive fish farming is accompanied by the increasing use of natural resources and chemicals. Marine aquaculture is undergoing rapid expansion, more than all other animal food-producing sectors [1,2]. A wide variety of pesticides such as antifouling compounds, biocides, herbicides, and insecticides are routinely applied to enhance production efficiency and animal welfare. However, direct emissions of micro-contaminants into seawater from fish farming facilities may cause contamination of the marine environment [3,4]. Many chemicals commonly used in aquaculture have not been evaluated for their effects on aquatic environments [5]. In aquatic systems, pesticides can be easily adsorbed by particulate organic matter due to their octanol-water partition coefficient (K_ow_), which can lead to bioaccumulation and biomagnification in aquatic organisms [6,7], which in turn affects the health of humans consuming fish [8,9]. Consequently, the European Framework Directive (2013/39/EU) established in 2013 [10,11] compromised the environmental quality standards (EQSs) for 45 priority substances in water and biota samples [12].

Sample pretreatment is a major step in chemical analysis, especially in the analysis of trace analytes in environmental samples [13]. Pretreatment methods for the analysis of pesticides commonly include liquid-liquid extraction (LLE) [14], liquid-phase microextraction (LPME) [15], solid-phase microextraction (SPME) [16], dispersive liquid-liquid microextraction (DLLME) [17], molecularly imprinted solid phase extraction [18], QuEChERS [19,20], and solid-phase extraction (SPE) [21,22]. The latter is the most frequently used procedure for trapping organic contaminants from environmental samples [23,24] and is the recommended procedure in several Environmental Protection Agency (EPA) methods [25]. However, SPE often suffers from cartridge plugging and the consumption of large amounts of toxic solvents during the elution step [26]. In addition, it is associated with excessive cost per sample [27].

To overcome these disadvantages, magnetic-solid phase extraction (MSPE) has been proposed [19,28]. It adopts magnetic particles as adsorbents, which endows some unique features in extraction and solves problems associated with SPE [29,30] such as low solvent consumption, simple and short operation time, and suitability for the extraction of polar and non-polar compounds in gas, liquid, and solid samples [31]. Owing to these advantages, MSPE has been widely used in many scientific fields, especially in analytical chemistry [32,33,34]. Among the various magnetic nanoparticles used, Fe_3_O_4_ nanoparticles and nanocomposites decorated with Fe_3_O_4_ nanoparticles are the most used because of their low toxicity to human health and the environment and their eco-friendliness [35,36].

Although such nanoparticles have been previously applied in environmental analysis, including a recent study by Kalaboka et al. [37], where they were used to determine pharmaceuticals and artificial sweeteners in wastewater, our study provides a distinct application and advancement in this field. Specifically, this study focuses on pesticide residues in marine aquaculture water, a matrix with higher salinity and unique organic content, that imposes different extraction challenges. Chemometric tools, such as Box–Behnken Design (BBD) and response surface methodology (RSM), were employed for the multivariate optimization of extraction conditions, enhancing the robustness and efficiency of the analytical method. To the best of our knowledge, this is the first study applying C18-functionalized magnetic nanoparticles in aquaculture water matrices using chemometric optimization followed by real-world Mediterranean case study locations.

## 2. Results and Discussion

### 2.1. Preliminary Experiments

First, an appropriate extraction solvent for the MSPE technique was selected to recover the studied compounds (spiked seawater samples). The results revealed that ethyl-acetate had the highest extraction efficiency (expressed as recovery) compared with methanol, *n*-hexane, and acetone.

The effect of stirring on analytes extraction was investigated in the range of 2000–3000 rpm. The results showed that the extraction efficiency increased as the stirring speed increased to 2500 rpm and then remained almost constant. Therefore, 2500 rpm was selected as the optimal value.

Furthermore, adjusting the pH may improve the extraction yield of the protonated compounds. In most cases, pH is adjusted to obtain the analyte in its neutral form. Values between 4 and 10 were studied to evaluate the effect of this variable on the extraction efficiency. Alkaline conditions (pH > 10) were not assayed because of the hydrolysis of some organophosphate pesticides [38]. Results showed that the maximum extraction yield was obtained at neutral pH. The pesticides showed maximum sensitivity at neutral pH. Based on these findings, the pH was not adjusted, and the experiments were conducted using seawater samples with a pH of 7.7.

### 2.2. Box–Behnken Design (BBD)

To evaluate the main factors affecting the extraction performance of the MSPE method, namely, the amount of sorbent (Fe_3_O_4_@SiO_2_@C18), the extraction, and elution time (expressed as recoveries), a three-level-three-factor Box–Behnken design (BBD) was applied. A total of 15 runs were carried out. Low, middle, and high levels of the coded values were designated for the variables as 1, 0, and −1, respectively. The coded and actual levels of the independent variables in the BBD experimental design matrix are listed in Table S1.

For this purpose, the STATISTICA 7.0 (StatSoft Inc., Tulsa, OK, USA) statistical package was used to generate the experimental matrix and to evaluate the results. A statistical analysis was performed to examine whether the experimental factors affected the performance of the proposed method. The main effects, interaction effects, and quadratic effects were evaluated at a spiked concentration of 50 μg L^−1^. Analysis of variance (ANOVA) and regression model analysis were performed, and a three-dimensional contour plot was drawn. A summary of the ANOVA results is presented in Table S2.

**Figure 1:**
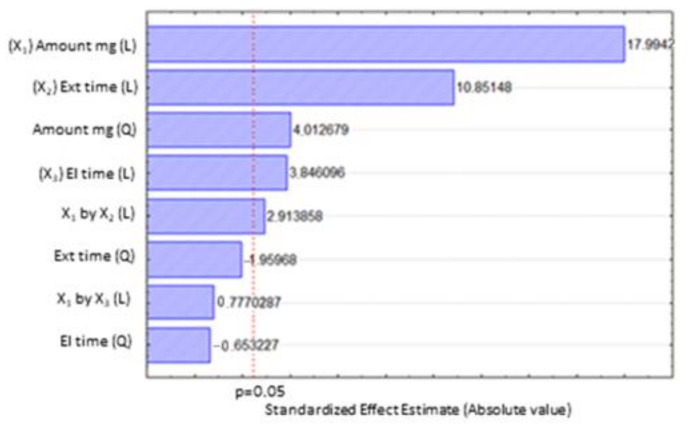
Standardized main effect Pareto chart for the Box-Behnken design. Vertical line in the chart defines 95% confidence level.

From the Pareto chart displayed in Figure 1, it can be observed that three linear term coefficients for the parameters (X_1_) amount of sorbent, (X_2_) extraction time, (X_3_) elution time, one interaction coefficient (X_1_ by X_2_) amount of sorbent by extraction time, and one quadratic effect for amount of sorbent (X_1_^2^) were significant. Data analysis at the 95% confidence level (*p* < 0.05) permitted an expression (Equation (1)) to be defined in terms of the significant coded factors:

Y (R%) = 57.75 + 16.375 X_1_ + 2.6875 X_1_^2^ + 9.87500 X_2_ +3.5 X_3_ + 3.75 X_1_ X_2_ (1)

The lack of fit, which measures the failure of the model to represent the data in the experimental domain at points that were not included in the regression, was also evaluated and shown to be insignificant (*p* value = 0.057). This fact indicates the good response of the model relative to the pure error, indicating a good response to the model.

**Figure 2:**
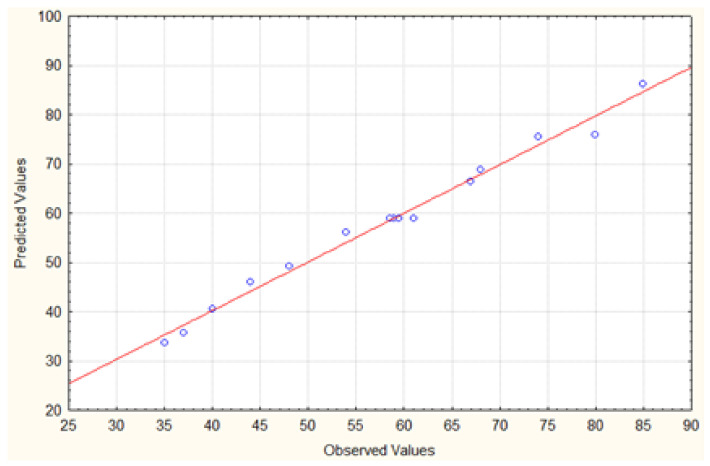
Plot of predicted versus actual values for the BBD design.

The experimental values plotted against the predicted responses for the extraction performance of the method displayed in Figure 2 showed a good determination coefficient (R^2^) of 0.98784, indicating that the model explained the experimental range studied well. The model regression coefficient is in reasonable agreement with the experimental results, indicating that 98.78% of the variability can be revealed by the model and is left with 1.22% residual variability. In addition, the adjusted correlation coefficient of 0.97162 was also high, indicating the high significance of the model.

**Figure 3:**
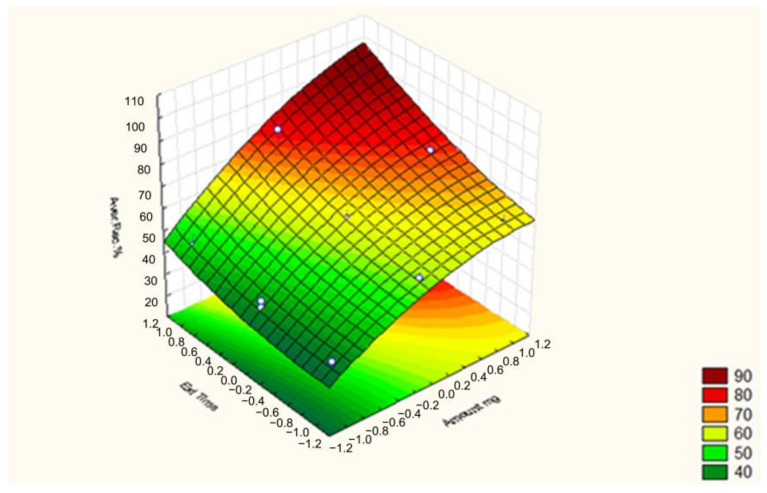
Response surfaces for the BBD-design between the absorbent amount and extraction time.

Based on results of the BBD, the overall interaction effects between the amount of sorbent and extraction time are displayed in Figure 3. Higher yields were obtained when higher amounts of sorbent were used for the extraction. The reflected interaction effects between the sorbent amount and extraction time demonstrated that the highest recovery of the target analytes was observed at the highest values for both variables.

**Figure 4:**
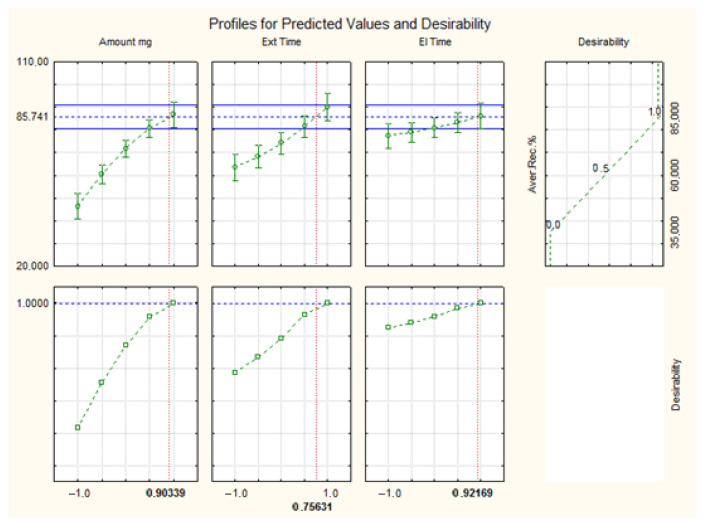
Profiles for predicted values and desirability for the optimal extraction conditions.

Finally, optimal extraction conditions were obtained using the desirability function of the statistical software (Figure 4). The optimal conditions for extracting pesticides from water samples were as follows: 54 mg of Fe_3_O_4_@SiO_2_@C18 as sorbent, no adjustment of pH, 5 min of extraction time, and 3 mL of ethyl acetate (2 × 1.5 mL) as elution solvent for an elution time of 4.5 min.

### 2.3. Analytical Performance and Method Validation

The figures of merit (Table 1) of the proposed method under the optimum extraction conditions established above were evaluated by determining parameters such as the limits of detection (LODs) and quantification (LOQs), intra-day and inter-day precision, accuracy expressed as absolute recovery, and the linear range.

The developed MSPE procedure demonstrated good linearity (from LOQ- 500 ng L^−^^1^), with coefficients of determination (R^2^) for the calibration curves between 0.9901 and 0.9999 for all analytes. LODs and LOQs were calculated from spiked aquaculture water samples with the target analytes at multiple levels and subjected to the full MSPE procedure. A typical chromatogram of a spiked sample is presented in Figure S1. The method LODs and LOQs were estimated based on signal-to-noise ratios of 3 (S/N ≥ 3) and 10 (S/N ≥ 10), respectively. Based on this, the LODs were obtained in the range of 1.9 to 30 ng L^−^^1^, with the exception of permethrin, for which a higher LOD was observed at 62 ng L^−^^1^. This also led to a higher LOQ (206 ng L^−^^1^), which restricted the linearity range of the calibration curve for this compound. This can be attributed to its strong hydrophobic character (log K_ow_ ~6.5), low volatility, and low ionization efficiency in GC–MS, all of which reduce the signal intensity at low concentrations. Additionally, matrix effects from aquaculture water and potential adsorption losses on glassware may have further contributed to this. The accuracy of the method was evaluated using recovery studies. Pesticide-free seawater samples were spiked at three concentration levels: LOQ, 5 LOQ, and 10 LOQ for each analyte. Recoveries based on three replicate extractions were evaluated in the range of 60% and 99%. Intra-day repeatability (RSDr%, *n* = 5) and inter-day reproducibility (RSD_R_%, *n* = 5 × 3 days) were determined by calculating the relative standard deviation (RSD). Under the optimized conditions, intra-day RSDs were equal to or lower than 15%, indicating an acceptable precision.

Compared to previous applications of such sorbents in water/wastewater systems [37,39,40,41], our method offers a new and environmentally relevant application for monitoring pesticide contamination in aquaculture water systems, which are increasingly recognized as potential sources of chemical residues in seafood. The chemometric approach enabled the accurate identification of optimal extraction conditions with fewer experimental runs, demonstrating the good efficiency of the method.

### 2.4. Reusability of Fe_3_O_4_@SiO_2_@C18

Repeatability is a key factor in evaluating sorbent performance. In order to investigate the recycling of the nanoparticle sorbents, the Fe_3_O_4_@SiO_2_@C18 sorbents used in the MSPE procedure were rinsed twice with 5 ml of ultra-pure water and 5 mL of acetone, and thoroughly dried with a stream of nitrogen each time before reuse. Subsequently, 54 mg of regenesis magnetic adsorbent was added to a beaker containing 10 mL of the seawater sample. Seven replicate analyses were performed according to the procedure described in a following paragraph (Section 3.5) at a concentration of 50 μg L^−^^1^ for each compound. The results obtained showed that the recoveries of chlorothalonil and λ-cyhalothrin were below 60% in the fourth cycle, while the rest of the compounds were above 60%. Therefore, the sorbent could be used at least three times. The RSDs of mean extraction recoveries were between 8 and 19%. These results indicate that the sorbents are stable and have no carry-over of analytes during the extraction procedure, showing good reusability.

### 2.5. Analysis of Target Compounds in Real Water Samples

The proposed method was successfully applied to the analysis of nine water samples collected from an aquaculture area of Thesprotia (N.W. Greece), Thermaikos gulf (N. Greece), and Butrint (S.W. Albania). In Thermaikos gulf, especially in estuaries of Loudias river, two insecticides were detected, namely, methyl-parathion (0.22 μgL^−^^1^) and chlorpyriphos (0.23 μgL^−^^1^), and one biocide irgarol at the concentration of 0.11 μgL^−^^1^. At the Thesprotia sample station, the concentration of irgarol was below the quantification limit.

## 3. Materials and Methods

### 3.1. Chemicals and Materials

Pesticides were obtained from Sigma-Aldrich (Steinheim, Germany) and were of high-purity grade (>99%). Table 1 lists the compounds studied, along with their main physicochemical properties. Individual stock standard solutions were prepared in methanol (1000 mgL^−1^) and stored in amber glass vials at −20 °C. Fresh working solutions and mixtures containing all pesticides were prepared by diluting the appropriate volumes of individual solutions.

Ferric chloride (FeCl_3_) was purchased from Fluka (Milwaukee, WI, USA), and ferrous chloride tetrahydrate (FeCl_2_·4H_2_O) from Ferak (Berlin, Germany). Tetraethyl orthosilicate (TEOS) and trimethoxy(octadecyl)silane (99%) were purchased from Sigma-Aldrich. Ammonia (NH_3_ 25%) was obtained from Merck (Darmstadt, Germany). All solvents used were of pesticide residue analysis grade and purchased from Labscan (Dublin, Ireland). Membrane filters (PVDF, 0.45 μm) were obtained from Millipore (Carrigtwohill, County Cork, Ireland). Seawater samples selected from the Thesprotia region (reference point), free from the analytes of concern, were used for method optimization and validation. All selected pesticides along with their physicochemical properties are visualized in Table S3 of the Supplementary Materials.

### 3.2. Instrumental Analysis

The analyses were performed using a Trace GC Ultra instrument (Thermo Scientific, Austin, TX, USA) coupled with an ISQ Mass Spectrometer controlled by a computer running the Xcalibur software. The separation was performed using a DB-5-MS column with a film thickness of 0.25 μm (30 m × 0.25 mm i.d.), Thermo Fisher Scientific. Helium (purity > 99.999 vol %, Air Liquid, Greece) was used as the carrier gas at a flow rate of 1 mL min^−1^. The GC oven temperature program was as follows: initial temperature of 60 °C (held for 1 min), 10 °C min^−1^ to 130 °C, 4 °C min^−1^ to 230 °C, and finally 8 °C min^−1^ to 250 °C (held for 10 min). The injector was set at 250 °C in splitless mode and the injection volume was 1 μL. The temperatures of the ion source and the MS transfer line were set to 230 °C and 280 °C, respectively. The mass spectrometer was operated in the electron ionization mode at an ionization energy of 70 eV. In the selected-ion monitoring (SIM) acquisition mode, the target ions were monitored at different time-windows, defined by the corresponding retention times. The quality criteria adopted for the retention times of the analytes, as well as the relative intensities of the selected ions, were within the tolerances established by the 2002/657/EC directive concerning the performance of analytical methods and the interpretation of results (2002/657/EC, 2002).

X-ray powder diffraction (XRD) measurements were performed using an X-ray diffractometer (D8 Advance Bruker, Germany) operating with Cu Kα (λ = 1.5406 Å). Elemental analysis of the Fe_3_O_4_@SiO_2_@C18 NPs was performed on a Vario Macro CNS (Elementar Analysensysteme GmbH, Langenslbold, Germany). Infrared spectra were obtained using a Fourier Transform-Infrared (FT-IR) Spectrometer (FT-IR Spectrum 100, PerkinElmer, Shelton, CT, USA) to identify the functional groups and chemical bonds of the coated materials. The samples were characterized using scanning electron microscopy (SEM) (JSM-5600, JEOL, Tokyo, Japan) with gold coating. The surface area of Magnetic NanoParticles (MNPs) was calculated based on N_2_ adsorption–desorption porosimetry using the BET method on an Autosorb-1 porosimeter (Quantachrome Instruments Inc., Boynton Beach, FL, USA). Before measurement, the samples were degassed at 80 °C for 5 h. Samples were vortexed with Vortex-IR (Starlab (Milton Keynes, UK), Ltd.) and sonicated with Elmasonic P (Elma Schmidbauer GmbH, Singen, Germany). Magnetic separation was assessed using a strong magnet with 1.4 T magnetic fields (10 cm × 5 cm × 4 cm).

### 3.3. Preparation of C18 Functionalized Magnetic Nanoparticles

The magnetic solid-phase extraction sorbent Fe_3_O_4_@SiO_2_@C18 was synthesized and characterized according to a previously reported method [34]. First, the MNPs of Fe_3_O_4_ were synthesized by the co-precipitation of Fe^2+^ and Fe^3+^ ions under alkaline conditions and hydrothermal treatment [32,42] and washed several times with deionized water and ethanol.

Next, a silica coat was fabricated on the surface of Fe_3_O_4_ MNPs using the Stöber process with minor changes [43,44,45]. Briefly, Fe_3_O_4_ MNPs (0.5 g) were dispersed in a mixture of ethanol (12 mL) and deionized water (4 mL). Then, 0.25 mL of tetraethyl orthosilicate (TEOS) and 0.5 mL ammonia 25% (*w*/*w*) were added dropwise to the mixture, followed by stirring under a nitrogen stream for 10 min. The reaction proceeded for 12 h. After washing several times with ethanol and water, the obtained Fe_3_O_4_@SiO_2_ MNPs were dried in a vacuum oven.

Finally, the Fe_3_O4@SiO_2_ MNPs were functionalized with trimethoxy(octadecyl)silane owing to their lipophilic character, which is desirable for the selected analytes. Specifically, 0.2 g silica-coated NPs were dispersed in 70 mL of anhydrous toluene with the aid of ultrasonication, followed by the addition of 0.2 mL trimethoxy(octadecyl)silane. The resulting mixture was then refluxed at 80 °C while stirring for 12 h. After cooling to room temperature, the obtained product (Fe_3_O_4_@SiO_2_ @C18) was rinsed several times with toluene and ethanol and dried under vacuum at room temperature, before use.

### 3.4. Characterization of MNPs

Given the different physicochemical properties of aquaculture water compared to wastewater, and the different analyte classes, structural confirmation was repeated to ensure sorbent integrity and reproducibility across matrices. The successful fabrication of Fe_3_O_4_ and functionalization with silica coat and C18 groups was confirmed by the IR spectra of Fe_3_O_4_ naked, Fe_3_O_4_@SiO_2_, and Fe_3_O_4_@SiO_2_@C18 magnetic nanoparticles. The spectrum of Fe_3_O_4_ nanoparticles showed a strong absorption band at 570 cm^−1^, which corresponded to the Fe-O-Fe vibration. The absorption peak at 1068 cm^−1^ can be attributed to the Si-O-Si vibration, while the peaks at 1634 and 3400 cm^−1^ can be assigned to the absorbed water or the silanol groups (Si-OH) of silica. The presence of absorption bands at 2928 and 2854 cm^−1^ corresponded to symmetrical and asymmetrical CH2 stretching vibrations in the -(CH_2_)_17_CH_3_ chain [19,46,47,48].

Elemental analysis of Fe_3_O_4_@SiO_2_@C18 NPs showed a carbon content of 7.05%, while the total specific surface area of Fe_3_O_4_@SiO_2_@C18 was found to be 151 m^2^/g using a BET plot.

The spinel structure of Fe_3_O_4_ was unchanged by the introduction of a silica coat and immobilization of the C18 group. The XRD patterns of Fe_3_O_4_@SiO_2_ and Fe_3_O4@SiO_2_@C18 were like those of Fe_3_O_4_ MNPs according to the six characteristic peaks appearing at 2θ of 30.1°, 35.5°, 43.1°, 53.4°, 57.0°, and 62.6° in all spectra.

The mean crystallite size (D, nm) of the particles was estimated using the Scherer equation:

D = (K λ)/(β cosθ)(2)

where K is a dimensionless shape factor, λ is the X-ray wavelength, β is the line broadening at half -maximum intensity, and Θ is the Bragg angle [46]. Based on the Scherer equation, the mean crystallite size of Fe_3_O_4_@SiO_2_@C18 was 8.4 ± 1.5 nm.

### 3.5. Magnetic Solid-Phase Extraction (MSPE) Procedure

The MSPE procedure was carried out as follows: First, 54 mg of MNPs was rinsed in 2 mL methanol and then dispersed into 10 mL seawater sample. Next, to trap the analytes, the mixture was shaken manually for 1 min, vortexed for 1 min, and sonicated for 5 min. Subsequently, Fe_3_O_4_@SiO_2_@C18 MNPs were isolated from the solution with a strong magnet at the bottom of the beaker and most of the supernatant was discarded. The residual solution and the MNPs were totally transferred to a 15 mL polypropylene centrifuge tube. The particles were aggregated again by positioning a magnet on the outside of the tube wall so that the residual solution could be completely removed with a pipette. The isolated particles were then vortexed twice with 1.5 mL of ethyl acetate and sonicated for 4.5 min to desorb the analytes. After positioning a magnet outside the centrifuge tube, the supernatant ethyl acetate solution was collected in a vial. The final extract was then dried over anhydrous sodium sulfate. The extracts were evaporated to dryness under a gentle stream of nitrogen, reconstituted in 0.1 mL of n-hexane, and stored at −20 °C prior to chromatographic analysis.

## 4. Conclusions

In summary, Fe_3_O_4_@SiO_2_@C18 magnetic sorbent was prepared by hydrothermal reaction to serve as a solid-phase extraction sorbent for the simultaneous enrichment and detection of pesticides used in aquaculture facilities. The optimization of the magnetic solid-phase extraction method (MSPE), as well as the investigation of the interaction effect of different factors, was achieved with a three-factor, three-level Box–Behnken design, response surface methodology, and desirability profile. Validation parameters confirm that the reported method can provide the necessary sensitivity, linearity, precision, and accuracy. The evaluation of sorbent reusability, combined with successful recoveries in real aquaculture samples, illustrates the practical utility of this method for routine environmental surveillance in the aquaculture industry. Moreover, this method is simple, reproducible using non-toxic or poisonous reagents, and is a promising alternative to standard SPE and LLE. The results indicate the suitability of the proposed analytical protocol for detecting pesticides in high-salinity water samples and could be applied to other trace organic pollutants in different water samples.

## Figures and Tables

**Table 1:** Quality factors for the analytical performance of MSPE.

Compound	R^2^	Linearity (ng L^−1^)	LOD (ng L^−1^)	LOQ (ng L^−1^)	RSD_R_ Inter (%)	Concentration Level					
						LOQ		5 LOQ		10 LOQ	
						R%	RSD_r_ Intra (%)	R%	RSD_r_ Intra (%)	R%	RSD_r_ Intra (%)
Ethoxyquine	0.9993	30–500	9.0	30	17	63	15	65	9.6	63	3.3
Atrazine	0.9995	27–500	8.1	27	12	85	11	83	8.2	88	3.9
Chlorothalonil	0.9901	87–500	26	87	14	62	14	63	9.0	60	3.6
Chlorpyriphos-methyl	0.9999	6.4–500	1.9	6.4	9.6	91	8.3	90	6.1	92	2.4
Methyl-parathion	0.9978	52–500	16	52	8.8	98	8.4	99	6.3	99	2.9
Chlorpyriphos	0.9901	69–500	21	69	9.9	96	8.2	95	6.6	97	2.3
Resmethrin	0.9909	31–500	9.5	31	17	67	15	67	8.9	64	4.1
λ-cyhalothrin	0.9931	90–500	27	90	17	60	15	60	9.1	61	4.0
Permethrin	0.9916	206–500	62	206	14	75	12	76	8.4	83	3.1
Irgarol	0.9996	99–500	30	99	12	84	10	84	7.9	85	3.8

## Data Availability

Data is unavailable due to privacy.

## Supplementary Materials

The following supporting information can be downloaded at: https://www.mdpi.com/article/10.3390/molecules30092076/s1, Table S1: Box–Behnken design matrix of three variables in coded units and the response of average extraction efficiency (R%); Table S2: ANOVA results obtained by BBD design; Table S3: Selected compounds and their physicochemical-environmental properties; Figure S1: GC–MS chromatogram of a spiked sample at concentration level of 5 LOQ; Table S4: Comparison of LODs and LOQs of the proposed method with other studies. Refs. [49,50,51,52,53,54,55,56] are cited in Supplementary Materials.

## Abbreviations

The following abbreviations are used in this manuscript:

MSPE	Magnetic Solid-phase extraction
GC–MS	Gas Chromatography–Mass Spectrometry
MNPs	Magnetic Nanoparticles
BBD	Box–Behnken design
