# Supplementary material for: Magnetic Solid-Phase Extraction Based on C18 Nanoparticles for the Determination of Pesticides in Aquaculture Water Samples
Source: Molecules. 2025 May 7;30(9):2076. doi: 10.3390/molecules30092076 (PMC12073648; doi:10.3390/molecules30092076)
Supplement: Supplementary file 1 [file molecules-30-02076-s001.zip › molecules-3546371-supplementary.pdf]

Supplementary Material

Magnetic solid-phase extraction based on C18 nanoparticles for the determination of pesticides in aquaculture water samples

Margarita Kapsi [1,2,3]*, Vasileios Sakkas [4], Vasiliki Boti [1] and Triantafyllos Albanis [1]

[1] Laboratory of Industrial Chemistry, Department of Chemistry, University of Ioannina, 45110 Ioannina, Greece; vboti@uoi.gr (V.B.); talbanis@uoi.gr (T.A.)
[2] Institute of Oceanography, Hellenic Centre for Marine Research, 70014 Gournes, Greece
[3] Department of Agriculture, Hellenic Mediterranean University, 71410 Heraklion, Greece
[4] Laboratory of Analytical Chemistry, Department of Chemistry, University of Ioannina, 45110 Ioannina, Greece; vsakkas@uoi.gr

* Correspondence: m.kapsi@hcmr.gr

Table S1. Box–Behnken design matrix of three variables in coded units and the response of average extraction efficiency (R %)

**Factors**		**Levels**		
		**Low (-1)**	**Zero (0)**	**High (+1)**
(X_1) Amount (mg)		20	40	60
(X_2) Extr. Time (min)		3	5	7
(X_3) El Time (min)		2.5	5	7.5
Runs	X_1	X_2	X_3	Average recovery (R%)
1	-1	-1	0	35.0
2	1	-1	0	61.0
3	-1	1	0	44.0
4	1	1	0	85.0
5	-1	0	-1	37.0
6	1	0	-1	67.0
7	-1	0	1	40.0
8	1	0	1	74.0
9	0	-1	-1	48.0
10	0	1	-1	68.0
11	0	-1	1	54.0
12	0	1	1	80.0
13	0	0	0	59.0
14	0	0	0	58.5
15	0	0	0	59.5

Table S2.. ANOVA results obtained by BBD design

Factor	Sum of Squares (SS)	Degrees of freedom (df)	Mean Squares (MS)	F-value	p-value
(X_1) Amount mg (L)	2145.125	1	2145.125	323.7925	0.000002
Amount mg (Q)	106.673	1	106.673	16.1016	0.007017
(X_2) Ext Time (L)	780.125	1	780.125	117.7547	0.000036
Ext Time (Q)	25.442	1	25.442	3.8403	0.097738
(X_3) El Time (L)	98.000	1	98.000	14.7925	0.008498
El Time (Q)	2.827	1	2.827	0.4267	0.537822
X_1 by X_2 (L)	56.250	1	56.25	8.4906	0.026845
X_1 by X3 (L)	4.000	1	4.000	0.6038	0.466662
Pure Error	39.750	6	6.625		
Total SS	3268.500	14			

Table S3. Selected compounds and their physicochemical- environmental properties

Compound	Chemical group	Formula	pKa	Log kow (pH=7, 20°C)	Log D (pH 7.4)	Log P	Solubility in water (mg/L)	Vapor pressure (mPa)
Atrazine	Herbicide, Triazine	$C_8H_{14}ClN_5$	4.2 & 14.48	2.60	2.20	2.20	35.0	0.04
Ethoxyquine	Fungicide, Quinoline	$C_{14}H_{19}NO$	5.15	3.10	3.00	3.39	60.0	0.35
Chlorothalonil	Fungicide, Chloronitrile	$C_8Cl_4N_2$	*	3.05	4.10	4.10	0.81	0.08
Chlorpyriphos methyl	Insecticide, Organophosphate	$C_7H_7Cl_3NO_3PS$	*	4.31	4.07	4.07	2.74	3
Methyl parathion	Insecticide, Organophosphate	$C_8H_{10}NO_5PS$	7.15	2.86	2.60	2.60	55.0	0.2
Chlorpyriphos	Insecticide, Organophosphate	$C_9H_{11}Cl_3NO_3PS$	*	4.96	4.78	4.78	1.05	0.43
Resmethrin	Insecticide, Pyrethroid	$C_{22}H_{26}O_3$	*	5.43	5.07	5.07	0.01	0.0015
λ-Cyhalothrin	Biocide, Pyrethroid	$C_{23}H_{19}ClF_3NO_3$	10.65	7.00	5.68	5.68	0.005	0.0002
Permethrin	Insecticide, Pyrethroid	$C_{21}H_{20}Cl_2O_3$	*	6.50	5.70	5.70	0.2	0.007
Irgarol	Biocide, Triazine	$C_{11}H_{19}N_5S$	6.68 & 14.13	3.99	2.91	2.99	7.0	0.09

Data predicted with www.chemspider.com and PPDB

(https://sitem.herts.ac.uk/aeru/ppdb/en/

*: No ionizable atoms found

Table S4. Comparison of LODs and LOQs of the proposed method with other studies

Compound	Chemical group	LOD (ng/L)	LOQ (ng/L)	Reference
Atrazine	Herbicide, Triazine	8.1	27	Proposed Method
		120	400	[49]
		40	120	[50]
		1250	3790	[51]
Methyl Parathion	Insecticide, Organophosphate	16	52	Proposed Method
		950	4520	[52]
		28	42	[53]
		83	250	[54]
Chlorpyriphos	Insecticide, Organophosphate	21	69	Proposed Method
		80	270	[55]
		1000	3400	[56]
		83000	310000	[53]

Figure S1. GC/MS chromatogram of a spiked sample at 5×LOQ concentration level

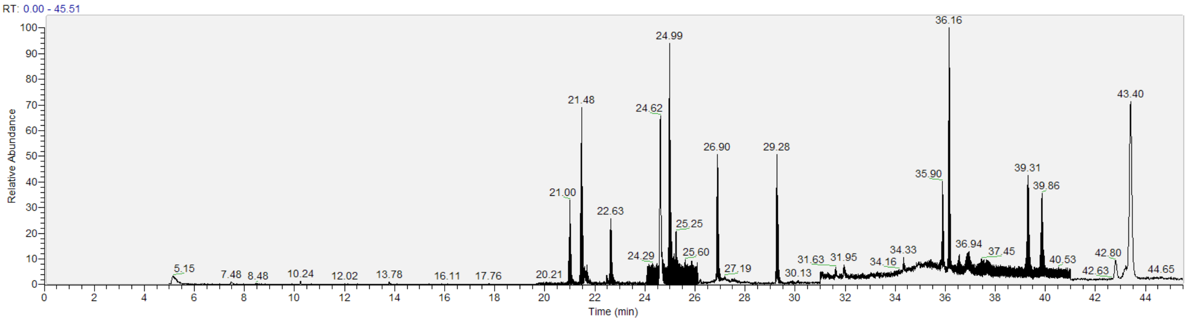

49. Bekele, H.; Megersa, N. Green Analytical Method Based on Salt Assisted Graphene Oxide Dispersive Solid Phase Extraction of Symmetrical Triazine Herbicides in Environmental Water Samples for Liquid Chromatographic Determination. *Int J Environ Anal Chem* 2024, doi:10.1080/03067319.2022.2161377.
50. Gabardo, R.P.; Toyama, N.P.; Amaral, B. do; Boroski, M.; Toci, A.T.; Benassi, S.F.; Peralta-Zamora, P.G.; Cordeiro, G.A.; Liz, M.V. de Determination of Atrazine and Main Metabolites in Natural Waters Based on a Simple Method of QuEChERS and Liquid Chromatography Coupled to a Diode-Array Detector. *Microchemical Journal* 2021, *168*, 106392, doi:10.1016/J.MICROC.2021.106392.
51. Prukjareonchook, A.; Alahmad, W.; Kulsing, C.; Chaisuwan, T.; Dubas, L. Selective Solid-Phase Extraction of Atrazine from Agricultural Environmental Water Samples Using High Permeability Nanoporous Carbon Derived from Melamine-Based Polybenzoxazine Followed by HPLC-UV. *Int J Environ Anal Chem* 2024, *104*, 2041–2055, doi:10.1080/03067319.2022.2056035.
52. Gil García, M.D.; Dahane, S.; Arrabal-Campos, F.M.; SocíasViciana, M.M.; García, M.A.; Fernández, I.; Martínez Galera, M. MCM-41 as Novel Solid Phase Sorbent for the Pre-Concentration of Pesticides in Environmental Waters and Determination by Microflow Liquid Chromatography-Quadrupole Linear Ion Trap Mass Spectrometry. *Microchemical Journal* 2017, *134*, 181–190, doi:10.1016/j.microc.2017.06.008.
53. Arnnok, P.; Patdhanagul, N.; Burakham, R. Dispersive Solid-Phase Extraction Using Polyaniline-Modified Zeolite NaY as a New Sorbent for Multiresidue Analysis of Pesticides in Food and Environmental Samples. *Talanta* 2017, *164*, 651–661, doi:10.1016/J.TALANTA.2016.11.003.

54. Pimenta, G.G.; De Queiroz, M.E.L.R.; Victor, R.P.D.; Noronha, L.M.; Neves, A.A.; De Oliveira, A.F.; Heleno, F.F. DLLME-GC/ECD Method for the Residual Analysis of Parathion-Methyl and Its Application in the Study of the UV-Photodegradation Process. *Article J. Braz. Chem. Soc* 2045, *28*, doi:10.21577/0103-5053.20170048.
55. Nasiri, M.; Ahmadzadeh, H.; Amiri, A. Organophosphorus Pesticides Extraction with Polyvinyl Alcohol Coated Magnetic Graphene Oxide Particles and Analysis by Gas Chromatography-Mass Spectrometry: Application to Apple Juice and Environmental Water. *Talanta* 2021, *227*, 122078, doi:10.1016/j.talanta.2020.122078.
56. Song, N.E.; Jung, Y.S.; Choi, J.Y.; Koo, M.; Choi, H.K.; Seo, D.H.; Lim, T.G.; Nam, T.G. Development and Application of a Multi-Residue Method to Determine Pesticides in Agricultural Water Using QuEChERS Extraction and LC-MS/MS Analysis. *Separations 2020, Vol. 7, Page 52* 2020, *7*, 52, doi:10.3390/SEPARATIONS7040052.
